# An objective *in vivo* diagnostic method for inflammatory bowel disease

**DOI:** 10.1098/rsos.180107

**Published:** 2018-03-21

**Authors:** Sophie C. Payne, Robert K. Shepherd, Alicia Sedo, James B. Fallon, John B. Furness

**Affiliations:** 1 Bionics Institute, East Melbourne, Victoria 3002, Australia; 2 Medical Bionics Department, The University of Melbourne, Parkville, Victoria 3010, Australia; 3 Department of Otolaryngology, The University of Melbourne, Parkville, Victoria 3010, Australia; 4 Department of Anatomy and Neuroscience, The University of Melbourne, Parkville, Victoria 3010, Australia; 5 Florey Institute of Neuroscience and Mental Health, Parkville, Victoria 3010, Australia

**Keywords:** inflammatory bowel disease, mucosal permeability, voltage transient, electrical impedance

## Abstract

Inflammatory damage to the bowel, as occurs in inflammatory bowel disease (IBD), is debilitating to patients. In both patients and animal experimental models, histological analyses of biopsies and endoscopic examinations are used to evaluate the disease state. However, such measurements often have delays and are invasive, while endoscopy is not quantitatively objective. Therefore, a real-time quantitative method to assess compromised mucosal barrier function is advantageous. We investigated the correlation of *in vivo* changes in electrical transmural impedance with histological measures of inflammation. Four platinum (Pt) ball electrodes were placed in the lumen of the rat small intestine, with a return electrode under the skin. Electrodes placed within the non-inflamed intestine generated stable impedances during the 3 h testing period. Following an intraluminal injection of 2,4,6-trinitrobenzene sulfonic acid (TNBS), an established animal model of IBD, impedances in the inflamed region significantly decreased relative to a region not exposed to TNBS (*p *< 0.05). Changes in intestinal transmural impedance were correlated (*p *< 0.05) with histologically assessed damage to the mucosa and increases in neutrophil, eosinophil and T-cell populations at 3 h compared with tissue from control regions. This quantitative, real-time assay may have application in the diagnosis and clinical management of IBD.

## 1. Introduction

Crohn's disease and ulcerative colitis, collectively known as inflammatory bowel disease (IBD), are debilitating episodic disorders of the gastrointestinal tract. IBD typically develops in young adulthood and affects patients throughout their lives [1,2]. Annual incidences of IBD are increasing, with 13–17 per 100 000 people being affected in the USA, and are associated with an annual direct care cost of $6.3 billion and productivity loss due to absenteeism of $3.6 billion [3]. The severity of the disease in patients is evaluated by symptomology, histology of biopsies, endoscopy and cytokine measurements [4,5]. Endoscopy is an indispensable tool for assessing the extent and severity of the inflammation and to determine responses to therapy, particularly mucosal healing, which is considered a primary indicator of remission from IBD [6,7]. Although endoscopy provides a visual assessment of gut inflammation, it needs to be supplemented by histological assessment of biopsy samples. Thus, a method that measures mucosal integrity without biopsy sampling would augment endoscopy findings.

The hallmark of active IBD is an infiltration of innate immune cells (neutrophils, macrophages and eosinophils) and adaptive immune cells (T cells and B cells) [8] into the intestinal mucosa. These immune cells secrete pro-inflammatory cytokines, such as interferon-γ [9] and TNF-α [10], activate intracellular mechanisms leading to the disruption of tight junctions, which regulate paracellular permeability [11]. Alterations to mucosal permeability are a key characteristic of IBD [12]. Changes in mucosal permeability can be examined clinically using non-invasive *in vivo* assays that measure the urinary excretion of large sugar molecules (e.g. lactulose) that are ingested and usually unable to cross the intestinal epithelium if the barrier is uncompromised [13]. However, permeability assays are time-consuming, influenced by confounding factors such as changes in gut transit and altered renal function, and vary considerably between individual patients, making the test hard to standardize [12,14]. Thus, this technique is seldom used for the evaluation of the severity of IBD [12].

**Figure 1.**
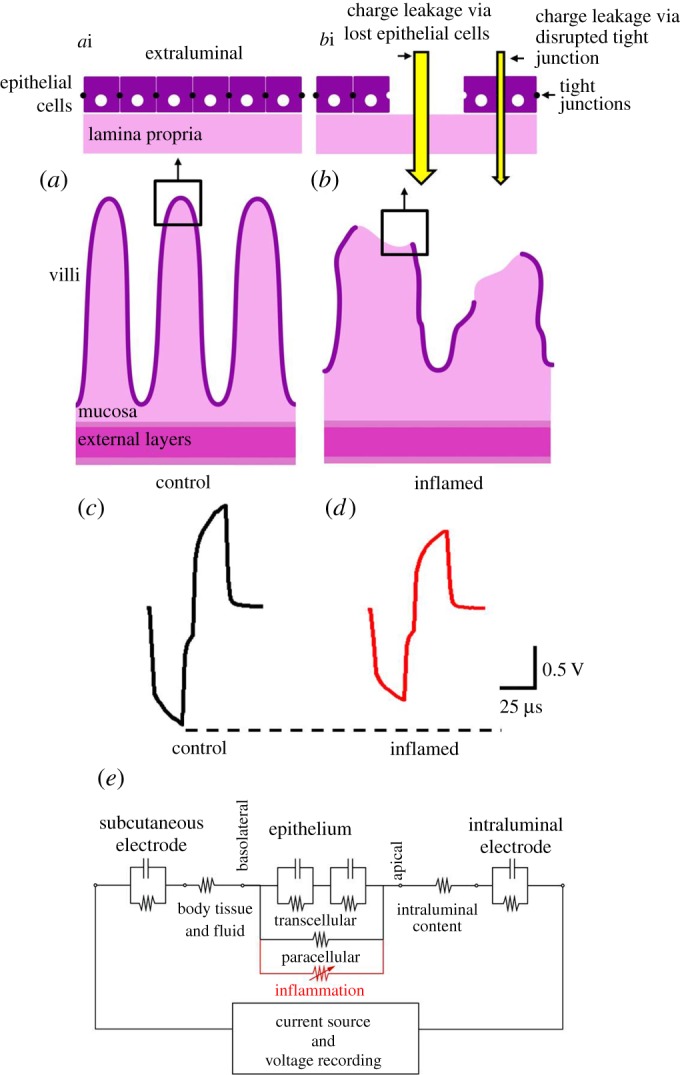
Schematic diagram of the wall of the intestine (*a*,*b*), responses to current pulses (*c*,*d*) and deduced equivalent circuit diagram (*e*). A schematic diagram of the control intestine (*a*) shows that cells of the epithelial cell layer (*a*i) are joined by tight junctions to form a high impedance barrier between the intraluminal space and the tissue of the lamina propria. Within inflamed gut tissue (*b*), epithelial cells are lost and/or tight junctions disrupted (*b*i), thereby allowing charge to flow through these pathways more readily. (*c*,*d*) Schematic examples of voltage transients induced across control (*c*) and inflamed (*d*) intestine, in response to a biphasic current pulse (25 µs per phase). The peak voltage transient (*V*_total_) is measured at the end of the first phase and converted into an impedance (*Z*_total_) using Ohm's Law. (*e*) The circuit diagram shows that the voltage measured *in vivo* is affected by impedances of the intraluminal electrode, intraluminal content, mucosal epithelium, body tissues and fluids, and the subcutaneous electrode. During inflammation, the epithelial impedance is reduced, resulting in a decrease in the peak voltage transient (*d*).

A number of animal models of IBD have been developed, the most common being rodents inflamed using 2,4,6-trinitrobenzene sulfonic acid (TNBS) [15–17]. In experiments using animal models of IBD, changes in ion permeability can be readily measured *ex vivo*, using explanted segments of intestine in an Ussing chamber [18–20]. In the Ussing chamber, voltage transients in response to applied currents are used to measure the transmucosal impedance [18,21]. In the present study, we have applied the principles of this technique *in vivo* and hypothesized that the inflammation-induced increase in transmucosal permeability due to the loss of epithelial cells and disruption of tight junctions would result in a decrease in gut wall impedance (figure 1). To evaluate the utility of electrical impedance to evaluate mucosal integrity, we have compared the changes in transmucosal impedance measured *in vivo* with assessment of inflammation by histopathology and immune cell infiltration.

## 2. Material and methods

### 2.1. Animals and surgical procedures

**Figure 2.**
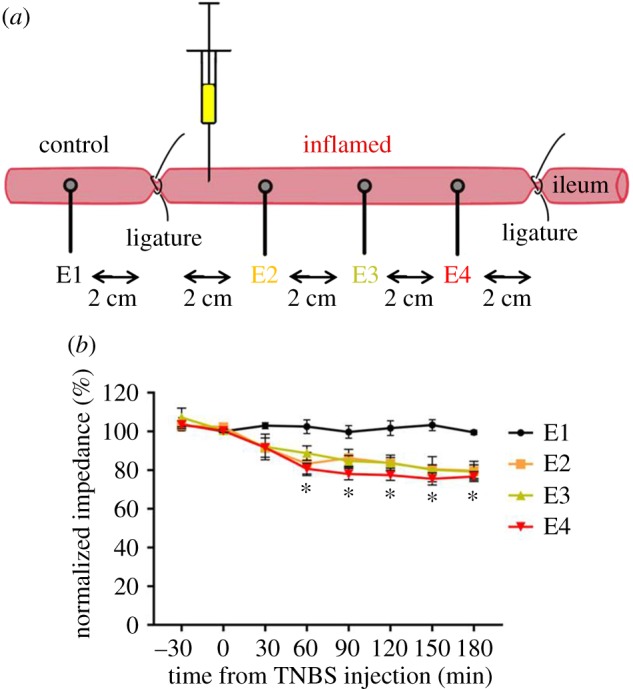
Effect of TNBS on transmucosal impedance. (*a*) Experimental arrangement. Electrode 1 (E1) was placed in a region of gut that did not receive TNBS (control), while the region containing electrodes E2–E4 was isolated using atraumatic ligatures and injected with TNBS. The voltage transient between each intraluminal electrode and a subcutaneous electrode return was measured. (*b*) Following TNBS injection, transmural impedance (normalized at *T* = 0) from inflamed regions significantly decreased between 60 and 180 min, compared with control. Data show mean (± s.e.m.), and differences were considered significant for *p* < 0.05.

All experiments used male Sprague–Dawley rats (*n* = 6; 10 weeks old; weight: 357 ± 17 g; Animal Resource Centre, Western Australia) and were approved by the Animal Research and Ethics Committee of the Bionics Institute. The experiments complied with the Australian Code for the Care and Use of Animals for Scientific Purposes (National Health and Medical Research Council of Australia) as well as the United States Army Medical Research and Material Command Animal Care and Use Review Office, protocol SSC-7486.02. Animals were allowed ad libitum access to standard chow, water and fresh food and were kept on a 12 L : 12 D cycle. Prior to surgery, animals were fasted overnight to reduce the amount of gut contents. On the day of surgery, rats were anaesthetized (2% isoflurane, 1% oxygen, flow rate of 1–1.5 l min^−1^; pre-operative analgesia carprofen 50 mg kg^−1^ subcutaneous), the abdominal cavity was opened and an 8 cm segment of jejunum was selected approximately 30 cm proximal to the ileo-caecal junction [22]. Four platinum (Pt) ball electrodes (1.2 mm diameter; exposed surface area 3.2 mm²), placed 2 cm apart, were inserted into the lumen and secured with sutures (7-0 silk, Ethicon; figure 2*a*). An 18 G needle placed subcutaneously acted as a return electrode. A 6 cm segment of the jejunum containing three Pt electrodes (E2–E4) was isolated using ligatures, while electrode 1 (E1) was placed 2 cm proximal to the ligated area. Following ligation, 30 min of baseline voltage transient measurements (see the next section for further detail) was generated from all electrodes (E1–E4). Inflammation was then induced within the ligated region by injecting TNBS (1 ml of 0.1% dilution in 50% ethanol, Sigma) into the lumen, similar to the procedure previously described [23,24].

### 2.2. Gut wall impedance monitoring

Gut wall impedance was monitored using biphasic current pulses passed between the intra- and extra-luminal electrodes. The peak voltage at the end of the first phase of the current pulse (total voltage *V*_total_, figure 1*c*,*d*) was measured as described previously [25]. A 25 μs per phase biphasic current pulse (7 μs interphase gap) was used to minimize the charging of the electrode–electrolyte interface. The current used in this study was 931 µA, which was chosen to produce a peak voltage transient of approximately 500 mV (range: 398–543 mV) when the Pt ball electrodes were placed in sterile 0.9% saline. The *V*_total_ value was then used to calculate total impedance (*Z*_total_) using Ohm's Law (*Z* = voltage/current). Following each acute experiment, the Pt ball electrodes were explanted, cleaned and retested in saline. There was no significant difference between pre- and post-experiment *in vitro* measures (paired *T*-test; *p* ≥ 0.05). Triplicate measurements were taken from each intraluminal electrode at *T* = −30 min and *T* = 0 prior to TNBS injection to establish a baseline. Following TNBS injection, triplicate measurements were taken at *T* = 30, *T* = 60, *T* = 90, *T* = 120, *T* = 150 and *T* = 180 min.

### 2.3. Histology and staining

Rats were euthanized (300 mg kg^−1^ Lethabarb, intracardial injection) at 3 h post-TNBS injection and segments of the jejunum adjacent to electrodes E1–E4 were removed, placed into cold PBS (0.15 M NaCl in 0.01 M sodium phosphate buffer, pH 7.2) and cut longitudinally along the mesenteric border, pinned out onto balsa boards and divided in half. Intraluminal content was gently removed by washing with cold PBS. One half was placed in fixative (2% formaldehyde plus 0.2% picric acid in 0.1 M sodium phosphate buffer, pH 7.4) overnight, embedded in paraffin, sectioned (5 μm), stained with haematoxylin and eosin (H&E) and mounted with DPX [23]. The other half of the tissue was prepared for myeloperoxidase (MPO) staining. Tissue was placed in ice-cold 100% ethanol (10 min), washed in cold 0.1 M sodium phosphate buffer (PB; 3 × 5 min) and then cryoprotected overnight (30% sucrose in PBS). Tissue was immersed overnight in 50% optimal cutting temperature medium and 50% sucrose, frozen (−20°C) and sectioned (14 µm). Sections were placed in freshly made Hanker–Yates solution (10 min in 0.003% H_2_O_2_ with 0.06 mg ml^−1^ Hanker–Yates reagent, Polysciences, Warrington, PA, USA, in PBS), washed then counterstained in methyl green aqueous solution (2% methyl green in distilled water for 30 s; Sigma), dehydrated (100% ethanol) and mounted with DPX.

### 2.4. Immunohistochemistry

Paraffin sections were dewaxed with xylene, graded ethanol and tap water. The sections underwent antigen retrieval (10 mM sodium citrate, 0.05% Tween 20, pH 6 and 1% hydrogen peroxide in PBS heated to 60°C for 10 min). Sections were incubated overnight at 4°C with anti-CD3, a cytotoxic T-cell marker (1 : 200 in 10% normal horse serum (NHS), Dako Cytomation). Sections were thoroughly washed (0.1 M PB) and the secondary antibody was applied (biotinylated goat anti-rabbit IgG 1 : 500 in 10% NHS, Dako E0432) for 1 h at room temperature. Following washing with 0.1 M PB, streptavidin coupled to horseradish peroxidase (1 : 1000 in 10% NHS, Dako P0397) was applied, and sections were washed (0.1 M PB) and immersed in 3,3′-diaminobenzidine (DAB) (made as per the instructions, Dako cat. no. #K3468) for 3 min. Slides were counterstained with Harris haematoxylin (5 s), washed (distilled water), dehydrated in 100% ethanol and xylene, and mounted in DPX.

### 2.5. Histopathology scoring

Histopathologist (J.B.Fu.), blinded to experimental conditions, used H&E-stained sections to evaluate the degree of inflammation at each electrode site (E1–E4). Histological changes were on a scale of 0–3 for the assessment of the extent and the degree of damage to the mucosa, and on a scale of 0–2 for assessment of the numbers of leucocytes within venules and the extent of haemorrhage within villi [26]. Scores were out of a total of 10 (table 1).

**Table 1.** Histological scoring table: parameters used to score inflammation on a scale from 0 to 10.

	**scoring criteria**	**score**
extent of mucosal epithelial damage	no damage	0
	damage affects less than one-third of villi	1
	damage affects between one-third and two-thirds of villi	2
	damage affects to more than two-thirds of villi	3
degree of mucosal damage	no damage	0
	swelling and loss of lamina propria matrix	1
	loss of surface epithelium from villus tips but bases of villi and residual villus cores present	2
	loss of villi to the level of the crypts	3
leucocyte presence in venules	venules have few leucocytes (as in normal tissue)	0
	venules contain four or more adherent leucocytes	1
	venules show numerous intravascular leucocytes	2
bleeding within villi	no bleeding within villi	0
	bleeding within villi common; no prominent blood clots formed	1
	bleeding within villi substantial; formation of blood clots	2

### 2.6. Cell counting

Eosinophilic granulocytes were identified morphologically using H&E-stained sections, by their distinctive nucleus and cytoplasmic staining [23] (identified by arrows in figure 4*a*,*b*). MPO activity was used to identify neutrophils (identified in figure 5*a*,*b* by arrows). Cytotoxic T-lymphocytes [27] were identified using antibodies to CD3 and counted in H&E sections (identified in figure 6*a*,*b* by arrows). With the observer blinded to the tissue identity, positive cells were counted with a ×40 objective, across 10 fields of view at each electrode position (E1–E4), using a Zeiss Axioplan II microscope, within the following layers: longitudinal smooth muscle, circular smooth muscle, submucosa and mucosa. Images of the total field of view were generated (Axiovision Software, Zeiss, Germany), the area of the total field of view for each layer of tissue was measured using ImageJ and the density of cells per square millimetre was calculated and analysed.

### 2.7. Statistical analysis

Gut wall impedance values (*Z*_total_) were normalized to the baseline measurements (average of data from *T* = −30 and *T* = 0 min prior to TNBS injection), set at 100%. A two-way repeated-measures analysis of variance (ANOVA) (Electrode × Time) was used to test for differences and interactions, and Tukey's post hoc test used where appropriate. Infiltration of leucocytes was assessed using a one-way ANOVA and a Tukey's post hoc test. Normalized gut wall impedances were correlated using with leucocyte infiltration and a Pearson's correlation coefficient (*R*) and two-tailed *p*-values generated. A *p*-value of <0.05 was accepted as statistically significant, and data are expressed as mean ± standard error of mean (s.e.m.). GraphPad Prism 4 was used for all analysis (GraphPad Software, USA).

## 3. Results

### 3.1. Decrease in gut wall impedance following intraluminal 2,4,6-trinitrobenzene sulfonic acid

Soon after implantation into the control jejunum, the *in vivo* impedance (*Z*_total_) for all electrodes was significantly elevated (1477 ± 158 Ω, *n* = 24 electrodes, *n* = 6 rats) compared with measurements made in saline (495 ± 37 Ω). *Z*_total_ *in vivo* is presumed to be dominated by the impedance of the gut wall, notably the impedance of epithelial cell barrier of the lining of the intestine, including the impedance provided by the tight junctions between epithelial cells (figure 1). An increase in transmural permeability is predicted to result in a drop in *Z*_total_ towards values measured in saline before implantation. Throughout the testing period, gut wall impedance in the non-inflamed region of the jejunum (at electrode 1) remained stable (*p* > 0.05; *n* = 6 rats, figure 2*a*,*b*). Following TNBS injection, gut wall impedances in inflamed regions (at electrodes E2–E4) rapidly decreased and were significantly less than the impedance measured at E1 at 60 min and remained so for the duration of the experiment (180 min; figure 2*b*; *p* < 0.05; *n* = 6). There were no significant differences between measurements recorded at the three electrodes in the inflamed region (E2, E3 and E4) following TNBS injection (*p* ≥ 0.05; figure 2*b*).

### 3.2. Histological scoring of inflammatory damage

**Figure 3.**
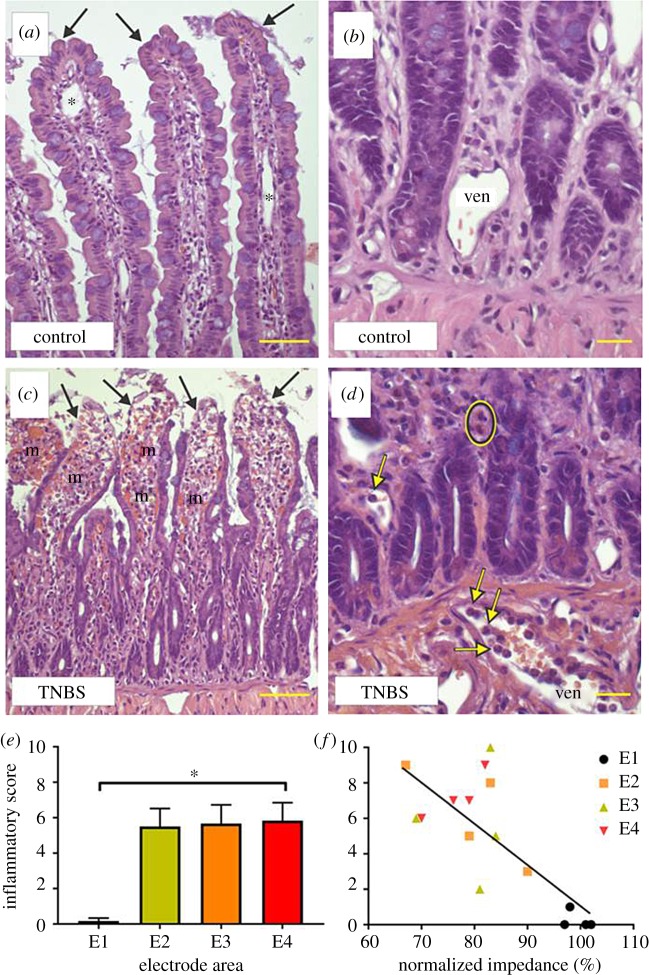
Inflammation-induced changes in the histological appearance of the jejunum following intraluminal TNBS injection. (*a*,*b*) The mucosa of the jejunum not exposed to TNBS. The surface epithelium was intact at the tips of the villi (arrows in (*a*)). Lymphatic vessels (asterisks) were apparent, but there were no red blood cells in the connective tissue spaces. The crypts were of normal appearance and immune cells were not accumulated in venules (ven). Following TNBS (*c*,*d*), the villi were shortened and the base of the mucosa can be seen in the same field as villus tips. The surface epithelium was lost from the tips of the villi (arrows). Numerous regions of microhaemorrhage were seen in the villi (m). After TNBS injection, there was marked accumulation of neutrophils in the venules (ven; indicated by arrows in (*d*)), which was not seen in control tissue (*b*). The crypts were intact in both control and TNBS-treated tissue. Eosinophils (circled) were more numerous in inflamed tissue (*d*), than in control (*b*). (*e*) Histologically, scored tissue damage was significantly more prevalent within inflamed tissue, than in control. (*f*) Inflammatory score of histological damage in the mucosa and normalized transmural impedance at 3 h post-TNBS injection is significantly correlated (*R* = −0.76). Data show mean (± s.e.m.), and differences were considered significant for *p* < 0.05. Scale bars in (*a*) and (*c*): 50 µm; (*b*) and (*d*): 20 µm.

In control tissue, villi were long and covered with intact surface epithelium (figure 3*a*, indicated by arrows), while venules had few to no inflammatory cells within them (figure 3*b*, indicated by ‘ven’). Three hours after infusion of TNBS, we examined the macroscopic appearance of the jejunum and observed patchy redness along the length of lumen exposed to TNBS, but not in other regions. Villi were damaged to various extents. Minimally damaged villi were swollen, and epithelial cells near the tip were flattened and fewer goblet cells were observed. More extensive damage of villi included loss of the surface epithelium from the tips and sides, but retention of the villus core (figure 3*c*, indicated by arrows). At the highest level of damage, the villus core had detached and the surface epithelium was lost from villus tips. Sloughed off epithelial cells were found in the lumen. There were many micro-haemorrhagic foci, consisting of clumps of extravasated red blood cells (figure 3*c*, indicated by ‘m’). These haemorrhages extended to the bases of the villi, but were rare in the region of the crypts. The crypt morphology was similar in samples from control and TNBS-treated jejunum, except in rare cases in which damage extended into the crypts. Venules in the submucosa and at the base of the mucosa after TNBS treatment contained numerous neutrophils and eosinophils (figure 3*d*, indicated by arrows). Quantitative scoring, based on parameters described in table 1, showed a significant difference between TNBS-exposed and control regions of the jejunum (*p *< 0.05; figure 3*e*). Inflammatory score of histological damage in the mucosa was significantly correlated to normalized transmural impedance at 3 h post-TNBS injection (*p* = 0.0006; *R* = −0.76; *n* = 16; figure 3*f*). There was no significant correlation between inflamed normalized gut wall impedance values generated from electrodes E2–E4 and inflammatory scores from these electrode areas (*p* = 0.394; *R* = −0.27; *n* = 12). No changes were observed in the external muscle layers. The histopathology reported in this study is similar to that described previously in experimental animals treated with TNBS [23].

### 3.3. Decrease in gut wall impedance correlated with immune cell infiltration

**Figure 4.**
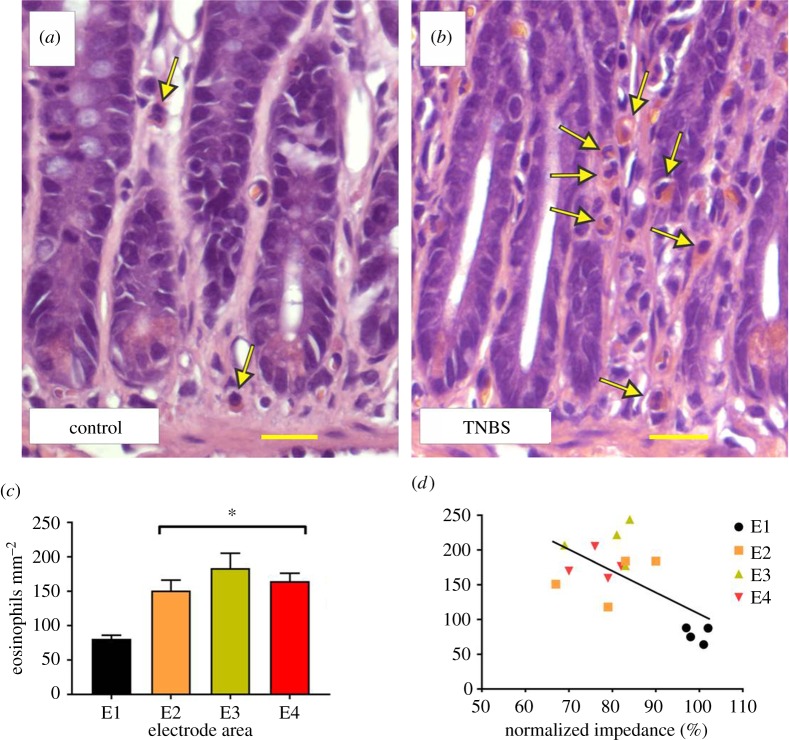
Infiltration of eosinophils into the mucosa correlates with the decrease in gut wall impedance following TNBS injection. (*a*,*b*) Eosinophils (indicated by arrows) were rarely seen in control mucosa layers (*a*), but were substantially more prevalent in inflamed mucosa tissue (*b*). (*c*) Quantitative data showing that significant infiltration of eosinophils into the mucosa occurred in inflamed tissue compared with control. (*d*) Eosinophil infiltration in the mucosa and normalized transmural impedance at 3 h post-TNBS injection is significantly correlated (*R* = −0.66). Data in (*c*) show mean ± s.e.m., and in (*d*) mean eosinophil density and normalized transmural impedance. Differences were considered significant for *p* < 0.05. Scale bar for (*a*) and (*b*): 20 µm.

Eosinophils were observed in small numbers within the mucosa (81 ± 5 cells mm^−2^) and submucosa (75 ± 18 cells mm^−2^) of control tissue (E1; *n* = 6 segments of control tissue; figure 4*a*,*c*). Very few eosinophils were observed in muscle layers (circular muscle: 2 ± 2 cells mm^−2^; longitudinal muscle: 0 cells mm^−2^). At 3 h following TNBS injection (figure 4*b*), the density of eosinophils was significantly greater within the mucosa of the inflamed segments of the jejunum (E2: 151 ± 15 cells mm^−2^; E3: 184 ± 22 cell mm^−2^; E4: 165 ± 12 cells mm^−2^; *n* = 6 rats; *n* = 18 pieces of jejunum; *p* = 0.001; figure 4*c*). No evidence of eosinophil infiltration within the submucosa and the longitudinal and circular muscle was found (*p* ≥ 0.05; *n* = 18, data not shown). There was a significant correlation between eosinophil infiltration into the mucosa and normalized gut wall impedance at 3 h post-TNBS injection (*p* = 0.006; *R* = −0.66; *n* = 16; figure 4*d*). However, there was no significant correlation between inflamed normalized gut wall impedance values generated from electrodes E2–E4 and eosinophil cell counts from these electrode areas (*p* = 0.500; *R* = 0.218; *n* = 12).

**Figure 5.**
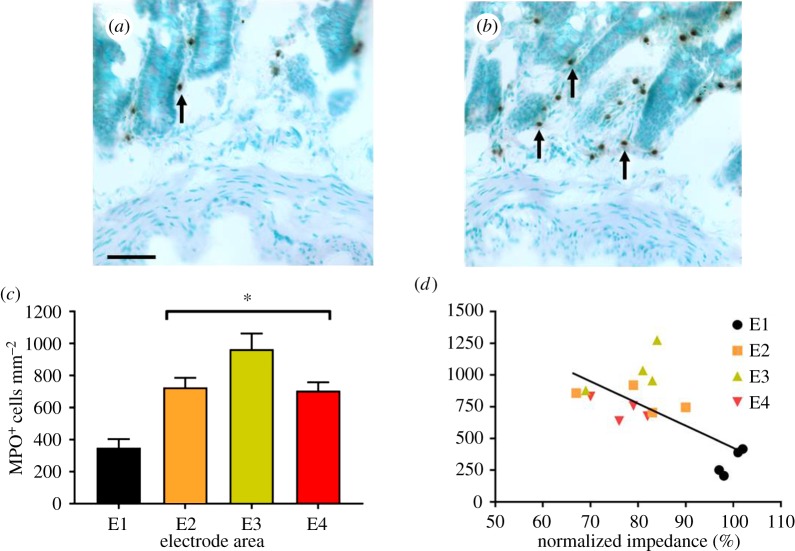
Infiltration of neutrophils into the mucosa correlates with decrease in gut wall impedance following TNBS injection. (*a*,*b*) Sections from control (*a*) and inflamed tissue (*b*) were counterstained with methyl green and neutrophils were identified by their MPO activity (MPO, indicated by arrows). (*c*) Following TNBS injection, the number of MPO-positive cells doubled within the inflamed mucosa at all electrode locations. (*d*) There was a significant correlation (*R* = −0.65) between normalized gut wall impedance and MPO density, across all electrode locations. Data in (*c*) show mean ± s.e.m. and in (*d*) mean MPO density and transmural impedance. Differences were considered significant for *p* < 0.05. Scale for (*a*) and (*b*): 50 µm.

Neutrophils were identified by their MPO activity (MPO+; figure 5*a*,*b*; indicated by arrows). MPO+ cells were observed in the smooth muscle layers (6 ± 2 cells mm^−2^), submucosa (217 ± 43 cells mm^−2^) and mucosa (350 ± 53 cells mm^−2^; figure 5*a*) of control tissue. Three hours after TNBS injection (*n* = 6 rats; *n* = 18 segments of inflamed jejunum), there was no infiltration of MPO+ cells into smooth muscle (*p* ≥ 0.05) or submucosa (*p* ≥ 0.05). However, there was significant infiltration of neutrophils into the mucosa (E2: 726 ± 60 cells mm^−2^; E3: 965 ± 98 cells mm^−2^; E4: 706 ± 52 cells mm^−2^; *p *< 0.0001; figure 5*b*), with no significant difference between neutrophil densities in different electrode locations of the region of TNBS infusion (E2, E3 and E4; figure 5*c*; *p* ≥ 0.05). The density of neutrophils in the mucosa was significantly correlated (*p* = 0.005; *R* = −0.65; E1: *n* = 4; E2–E4: *n* = 12) with normalized gut wall impedance measurements at 3 h post-TNBS injection (figure 5*d*). However, there was no significant correlation between inflamed normalized gut wall impedance values generated from electrodes E2–E4 and MPO+ cell counts from these electrode areas (*p* = 0.793; *R* = 0.085; *n* = 12).

**Figure 6.**
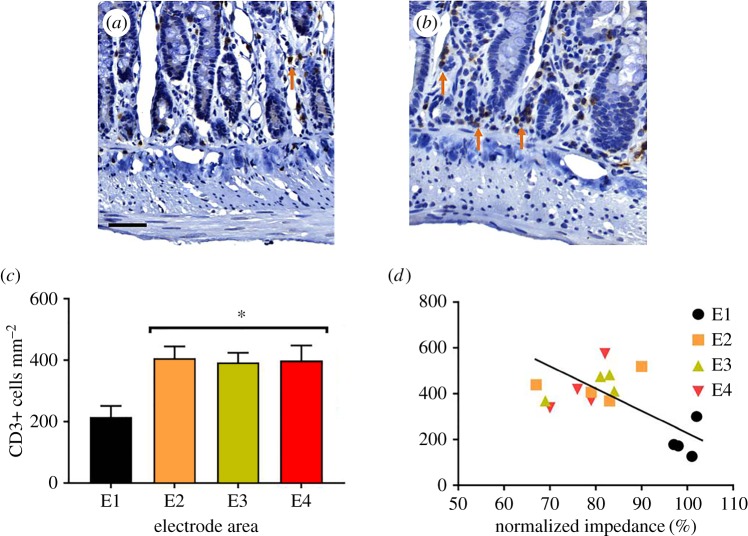
Increased T-cell numbers in the mucosa correlate with decreases in gut wall impedance following TNBS injection. (*a*,*b*) CD3+ cells were identified in haematoxylin counterstained sections (indicated by arrows) in control (*a*) and inflamed (*b*) tissue. (*c*) Following TNBS injection, the number of CD3+ cells doubled within inflamed mucosal tissue from all electrode locations. (*d*) There was a significant correlation (*R* = −0.57) between transmural impedance and CD3+ cell density. Data in (*c*) show mean ± s.e.m., and in (*d*) mean CD3+ cell density and transmural impedance. Differences were considered significant for *p* < 0.05. Scale bar, 50 µm.

CD3+ T cells were seen in control tissue (figure 6*a*; E1; *n* = 6 segments of control tissue; mucosa: 215 ± 36 cells mm^−2^; submucosa layer: 41 ± 14 cells mm^−2^; smooth muscle: 2 ± 1 cells mm^−2^). Following TNBS injection (figure 6*b*; *n* = 18 sections of inflamed jejunum), no changes occurred in numbers of CD3+ cells in the smooth muscle layer or the submucosa (*p* ≥ 0.05), while a significant increase in numbers of CD3+ cells was evident in the mucosa (E2: 407 ± 38 cells mm^−2^; E3: 393 ± 32 cells mm^−2^; E4: 399 ± 49 cells mm^−2^; *p* = 0.008; figure 6*c*). The densities of CD3+ cells were similar at electrode locations E2, E3 and E4 (figure 6*c*; *p* ≥ 0.05). The density of CD3+ cells in the mucosa was significantly correlated (*p* = 0.023; *R* = −0.57; E1: *n* = 4; E2–E4: *n* = 12) with normalized gut wall impedance measurements at 3 h post-TNBS injection (figure 6*d*). However, no correlation was seen between inflamed normalized gut wall impedance values (E2–E4) and CD3+ cell counts from these electrode areas (*p* = 0.087; *R* = 0.514; *n* = 12).

## 4. Discussion

Current clinical diagnostic and assessment methods for IBD include subjective, time-consuming and/or non-specific symptomology, serological, endoscopic and histological scoring, suggesting that there is value in an additional method to characterize disease activity that is real time and objective. We have developed transmural impedance as an *in vivo* technique that provided real-time quantitative data during experimentally induced inflammation of the jejunum. The decrease in transmural impedance correlated with the increase in T cells, neutrophil and eosinophil numbers, the quantification of which is a reliable and accepted measurement of inflammation. Therefore, the change in transmural impedance reported here can provide an *in vivo* indicator of mucosal integrity that can be measured in real time.

Stable control impedance values were generated from an electrode in an unchallenged region of rat small intestine over the entire 210 min experiment. Following TNBS injection, a rapid decrease in impedance occurred which was correlated with inflammatory damage that was assessed by histology or immune cell infiltration. The TNBS-induced model of jejunitis is an acute model of inflammation and does not fully represent the changes that occur in chronic IBD. That aside, our data support the measurement of transmucosal impedance as a marker for TNBS-induced inflammation.

The technology required to measure the induced voltage transients is standard for many neural prostheses and has a proven long-term safety record [28]. Moreover, the simplest implementation of the system requires only two electrodes (one intraluminal and one extraluminal). Such simplicity lends itself to integration into existing devices such as endoscopes. Thus, quantitative gut wall impedance data could be generated during endoscopy examinations, an idea that has recently been patented [29], and related to qualitative physician scoring of inflammation by visual inspection. In future studies, we aim to evaluate this technology in a clinical setting.

Assessment of mucosal integrity might have diagnostic application in other conditions, such as non-alcoholic fatty liver disease [30], which is characteristically associated with increased permeability of the duodenum, in non-erosive reflux disease (NERD) or in coeliac disease, in which a proportion of patients experience mucosal integrity disruptions and epithelial cell damage without visible signs of macroscopic alteration [31–33]. Because endoscopic abnormalities are not accurate indicators of NERD or coeliac disease, an objective measure that is undertaken in real time during the endoscopic procedure, without introducing additional risk, could augment current clinical assessment. The method may also have applicability to the assessment of IBD patients who are in endoscopic remission.

A second application for this technology is the longitudinal recording of real-time measurements of gut inflammation for closed-loop neuromodulation. A number of animal studies demonstrate that vagal nerve stimulation is a feasible, effective therapy for IBD that could mitigate risks associated with pharmacological intervention [17,34–36]. Neuromodulation of the vagus nerve may be preferable in patients who experience adverse side effects to common pharmaceutical therapies such as non-steroidal anti-inflammatory agents, corticosteroids and/or immunosuppressant drugs [37,38]. Owing to the episodic nature of IBD, in which patients can experience inflammatory episodes several times a year to once every 4 years [1], therapy is often administered to patients longer than is clinically required, thus risking side effects. Precise delivery of stimulation, while minimizing adverse side effects, is desirable for optimal therapy. The measurement described here is a biomarker of gut inflammation that can provide real-time information required for closed-loop control of vagal nerve stimulation in the treatment of IBD.

Other methods of determining tissue impedance exist, including electrochemical impedance spectroscopy and four-electrode impedance techniques [39,40]. While these techniques can provide rich data about the conductance of the tissue, they are typically more challenging to implement clinically and would require additional hardware, either in the form of additional electrodes (for four-electrode impedance measurements) or dedicated hardware for the generation and recording of sinusoidal waveforms (for electrochemical impedance spectroscopy). By contrast, the technique described here of monitoring voltage transients uses technology that is available in contemporary neural stimulators.

In conclusion, we have developed an objective, real-time measure of gut permeability that can be used to assess gastrointestinal inflammation *in vivo*. We consider this technology and methodology has translatable application in the evaluation of IBD.

## Data accessibility

Our data is available in an external publically shared folder in Dryad (http://dx.doi.org/10.5061/dryad.77bn3) [41].
